# Ocular surface disorders associated with the use of dupilumab based on WHO VigiBase

**DOI:** 10.1038/s41598-021-93750-3

**Published:** 2021-07-12

**Authors:** Sunny Park, Jung Hyun Lee, Ji Hyun Park, So Hyang Park, Song Yi Park, Yong Woo Jung, Soo An Choi

**Affiliations:** 1 College of Pharmacy, Research Institute of Pharmaceutical Sciences, Korea University, Sejong, South Korea; 2 College of Pharmacy, Korea University, Sejong, South Korea; 3 College of Pharmacy, DukSung Women’s University, Seoul, South Korea

**Keywords:** Health care, Pathogenesis, Medical research, Epidemiology

## Abstract

Dupilumab is a dual inhibitor of interleukin-4 and interleukin-13 and is mainly used to treat moderate-to-severe atopic dermatitis. Post-marketing safety data related to dupilumab have been accumulated, and it has been found that ocular surface diseases are closely associated with dupilumab treatment. The aim of this study was to detect dupilumab-related signals and to determine the safety characteristics of dupilumab with respect to eye disorders using real-world big data. Data on dupilumab use until December 29, 2019 were collected. The data were mined by calculating three indices: proportional reporting ratios, reporting odds ratios, and information components. The detected signals were classified using the primary system organ class in MedDRA terminology. Among 21,161,249 reports for all drugs, 20,548 reports were recorded for dupilumab. A total of 246 signals in the preferred terms were detected for dupilumab. Among the 246 positive signals obtained, 61 signals were related to eye disorders, which accounted for the largest percentage (24.8%), and 38 signals were anatomically related to the ocular surface. Dupilumab may cause extensive eye disorders; however, the underlying mechanisms and risk factors remain unclear. Our findings may facilitate broad safety screening of dupilumab-related eye disorders using real-world big data.

## Introduction

Atopic dermatitis (AD) is a chronic inflammatory condition caused by impaired immune system or genetic predispositions, resulting in hypersensitivity reactions in the skin and mucous membranes, following antigen interaction^1^. AD is characterized by increased sensitization to IgE and increased immunological activities of Th2, leading to the production of interleukin (IL)-4, IL-13, IL-5, IL-31, and IL-10 and causing intense pruritus, xerotic skin, erythema, edema, erosion, and lichenification. AD is not a serious disease; however, it is not easily treated and may lead to secondary infections that decrease patients’ quality of life (QoL). This decrease in QoL caused by AD is also associated with an increase in suicidal tendencies in patients with AD^2^. Therefore, systemic immunosuppressants, such as methotrexate, cyclosporine, and oral corticosteroids, are often prescribed for patients with moderate to severe AD^3^. Unfortunately, approximately 20% of patients with moderate to severe AD have limited or no clinical response to treatments approved by the FDA^4^. Significant side effects due to drugs (hypothalamus–pituitary–adrenal axis suppression), diabetes, osteoporosis, renal or liver toxicity, and myelosuppression have also been reported^5^.

Biologics have several advantages over conventional medications, such as target specificity and few side effects, but are associated with excessive pharmacological effects^6^. In patients with moderate to severe AD that cannot be adequately managed with systemic medications, dupilumab is indicated for treatment, with or without topical corticosteroids^7^. Dupilumab is a human monoclonal IgG antibody that binds to the IL-4Rα subunit, which is shared by the IL-4 and IL-13 receptor complexes, thereby inhibiting IL-4 and IL-13 signaling^7^. IL-4 and IL-13 play key roles in the development of AD symptoms, namely, decreased integrity and barrier function of the skin and level of peptides associated with antibiotics, which further cause skin barrier abnormalities such as pruritus, xerosis, blister, pigmentation, and lichenification^8^. In March 2017, dupilumab received approval for use in the USA as the first biologic for the treatment of AD^9^. Patients with AD who received dupilumab had clearer skin and experienced an alleviation in pruritus and sleeping problems, along with an improved QoL, after 16 weeks of treatment^10,11^. In addition, the expression of Th2 biomarkers and genes related to the activation of T cells were reduced after dupilumab treatment, resulting in an improved genetic profile associated with skin barrier function^12^. Unlike conventional treatments for AD, clinical safety trials have shown that dupilumab is associated with mild adverse events (AEs), such as conjunctivitis, keratitis, herpes zoster, hypersensitivity reactions, increased eosinophil count, and immunogenicity^11^. In particular, a higher incidence of conjunctivitis and keratitis was observed in phase 3 data, with one serious event^11^.

While premarketing clinical trials are short in duration and are carried out on a limited number of subjects, post-marketing surveillance involves a diverse population and provides comprehensive information about the drug. Therefore, safety considerations after the integration of post-marketing information would be important, especially for medications with unpredictable pharmacokinetic and pharmacodynamic properties, such as biologics^13^ or newly introduced medications with undefined safety profiles. For post-marketing safety data, VigiBase is the universal World Health Organization (WHO) global database of individual case safety reports (ICSRs) submitted by spontaneous AE reporting; VigiBase collects, assesses, and analyzes AEs. Spontaneous AE data are considered valuable; more than 60% of safety information was obtained from spontaneous AE reports in the European Medicines Agency from July 2012 to December 2013^14^. In particular, there was a case where the FDA added a boxed warning for tuberculosis on the approved labeling of infliximab in 2001, based on post-marketing data^15^.

Recently, several dupilumab-related eye disorders have been reported in patients with AD in the real world after the approval of the drug^16,17^, and the incidence of conjunctivitis has been found to be higher than that in clinical trial settings^18^. However, studies based on real-world big data in post-marketing settings have not been published. Thus, the aim of this study was to collect information related to dupilumab and to determine its safety characteristics and association with eye disorders using real-world big data.

## Materials and methods

### Data source and statistical analysis

For this study, AE data related to dupilumab were collected from the Uppsala Monitoring Center VigiBase, which comprised ICSRs from the member countries of the WHO Programme for International Drug Monitoring since 1968. All related and interacting AE reports that were collected until December 29, 2019 were used as source. The reports were submitted by regional physicians, pharmacists, and other health care professionals, as well as the public. The ICSRs included a unique number identifying each report, the date when the report was first entered in VigiBase, the continent of the primary source, reporters, age, gender, drug name, indication, seriousness, and name of the AE as coded by the Medical Dictionary for Regulatory Activities (MedDRA) terminology. The study protocol was designed in accordance with the relevant guidelines and approved by the Institutional Review Board of Korea University (IRB No. 2020–0208). The requirements for informed consent were waived by the board.

Basic demographic characteristics, including the year of report, region of report, reporter, age, and sex were analyzed. The year of report was when the report was first entered into VigiBase. The age at the time of onset of reaction/event was determined and categorized into seven groups: under 2 years, 2–11 years, 12–17 years, 18–44 years, 45–64 years, 65–74 years, and > 75 years. Reporters included physicians, pharmacists, other healthcare professionals, and consumers/non-health professionals. All statistical analyses were performed using SAS statistical application program (Version 9.4, SAS Institute Inc., Cary, NC, USA) and Microsoft Excel Software (2016).

### Data mining approach and signal detection criteria

Data mining involves finding hidden patterns or unforeseen associations from a large database using a computerized algorithm based on a measure of disproportionality. WHO defines signals as reported information on a possible causal relationship between an AE and a drug, the relationship being unknown or previously incompletely documented. To determine signals, a two-by-two contingency table of drug–AE co-occurrence was constructed as shown in Table 1, with the number of reports of the co-occurrence of interest.

**Table 1:** Two-by-two contingency table for analysis.

Number of reports	Specific adverse events (AEs)	All other AEs
Dupilumab	A	B
All other drugs	C	D

A: the number of reports containing both dupilumab-related and specific AEs, B: the number of reports containing dupilumab-related AEs but with all other AEs; C: the number of reports containing specific AEs but with all other drugs; D: the number of reports containing all other drugs and all other AEs.

Disproportionality analysis is the classical approach for signal detection in large databases, involving the calculation of the observed-to-expected events ratio. The most commonly used methods for disproportionality analysis are proportional reporting ratio (PRR)^19^ and reporting odds ratio (ROR) methods^20^. The estimates of the two methods are easy to calculate; however, the results are unstable with limited number of events^21^. To overcome this instability, Bayesian techniques were developed to adjust for uncertainty in the data by shrinking the estimates, including the information component (IC) based on the Bayesian Confidence Propagation Neural Network^22^. In this study, both frequentist and Bayesian methods were used, including ROR, PRR, and IC^19,22^. The PRR was defined as the ratio between the frequency of a specific AE reported for the drug of interest and the frequency of the same AE reported for all drugs in the comparison group. The ROR is the odds ratio between one specific AE reported and all other events for a given drug compared with the odds ratio for all other drugs in the database. The IC shows the quantitative dependency between the AEs and the drug^22^, and it is used to measure the disproportionality between the observed and expected reporting of the drug–AE combinations. The IC_025_ value is the lower limit of the 95% credibility interval for the IC. In this study, we used all three indices (PRR, ROR, and IC_025_) to assess the AE signal. AEs satisfying all predefined criteria were considered positive signals, as shown in Table 2^23^.

**Table 2:** Formula and criteria for signal detection.

Indices	Definition	Criteria for signal
PRR	{A/(A + B)}/{C/(C + D)}	≥ 2
ROR	(A/B)/(C/D)	≥ 2
IC	Log_2_P(AE,Drug)/P(AE)P(Drug)	Under limit of 95% CI ≥ 0

*RRR* proportional reporting ratio, *ROR* reporting odds ratio, *IC* information component, *AE* adverse event, *CI* credibility interval.

### Hierarchy analysis and anatomical classification

MedDRA terminology, which is the global standard for recording AEs and medical history^24^, was adopted. It has a hierarchical structure with five levels of sub-categories: system organ class (SOC), followed by high-level group term (HLGT), higher-level term (HLT), preferred term (PT), and lowest-level term (LLT)^25^. We used PTs of MedDRA version 23.0 and performed a hierarchy analysis of the detected signals to detect the HLT, HLGT, and primary SOC. Because MedDRA terminology has multiple axiality, a PT can be represented in more than one SOC. In this study, only the primary SOC was applied as the highest level of MedDRA hierarchy. The detected signals were graphically visualized using R Studio version 4.0.3.

The ocular surface consists of the surface and glandular epithelia of the cornea; conjunctiva; lacrimal gland, accessory lacrimal glands, Meibomian gland, and their apical and basal matrices; eyelashes with their associated glands of Moll and Zeis; and nasolacrimal duct^26^. Any disorder associated with these structures can be categorized as ocular surface disease (OSD)^27^. The detected signals of SOC eye disorders focused on the ocular surface were classified as “conjunctival,” “corneal,” “lid,” “lash,” and “lacrimal” based on the MedDRA terminology hierarchy. Signals containing “conjunctival,” “corneal,” “lid,” “lash,” or “lacrimal” in their HLT or PT level were regarded as anatomical OSD-related signals. For broader screening of ocular surface-related AEs caused by dupilumab, positive signals were subdivided into anatomic lines, including the secondary SOC and the primary SOC.

### Ethics approval

The study protocol was approved by Korea University’s Institutional Review Board (IRB No. 2020–0208).

### Consent to participate

The informed consent was waived by the board.

## Results

### Characteristics of dupilumab-related AE reports

The characteristics of dupilumab-related AEs reports are shown in Table 3. A total of 21,161,249 reports for all drugs were analyzed, out of which 20,548 reports were for dupilumab. Among the 20,548 reports, 18,372 were from the American continents, which constituted 89.41% of the reports, followed by Europe (9.74%). Dupilumab-related reports were largely reported in 2019 (78.33%). The main age groups of the patients were 18–44 years (5220 patients; 25.4%) and 45–64 years (4,852 patients; 23.61%). More than half were females (54.9%), and males accounted for 38.8%.

**Table 3:** Demographic characteristics of dupilumab-related adverse events.

	Characteristics	Number of dupilumab-related adverse events	%
Region of report	Americas	18,372	89.41
	Europe	2002	9.74
	Asia	149	0.73
	Oceania	25	0.12
Year of report	2016	3	0.01
	2017	58	0.28
	2018	4391	21.37
	2019	16,096	78.33
Age	< 2 years	11	0.05
	2–11 years	164	0.8
	12–17 years	704	3.43
	18–44 years	5220	25.4
	45–64 years	4852	23.61
	65–74 years	1201	5.84
	≥ 75 years	629	3.06
	Unknown	7767	37.8
Gender	Female	11,271	54.85
	Male	7979	38.83
	Unknown	1298	6.32

### Detected dupilumab signals in terms of primary SOC

**Figure 1:**
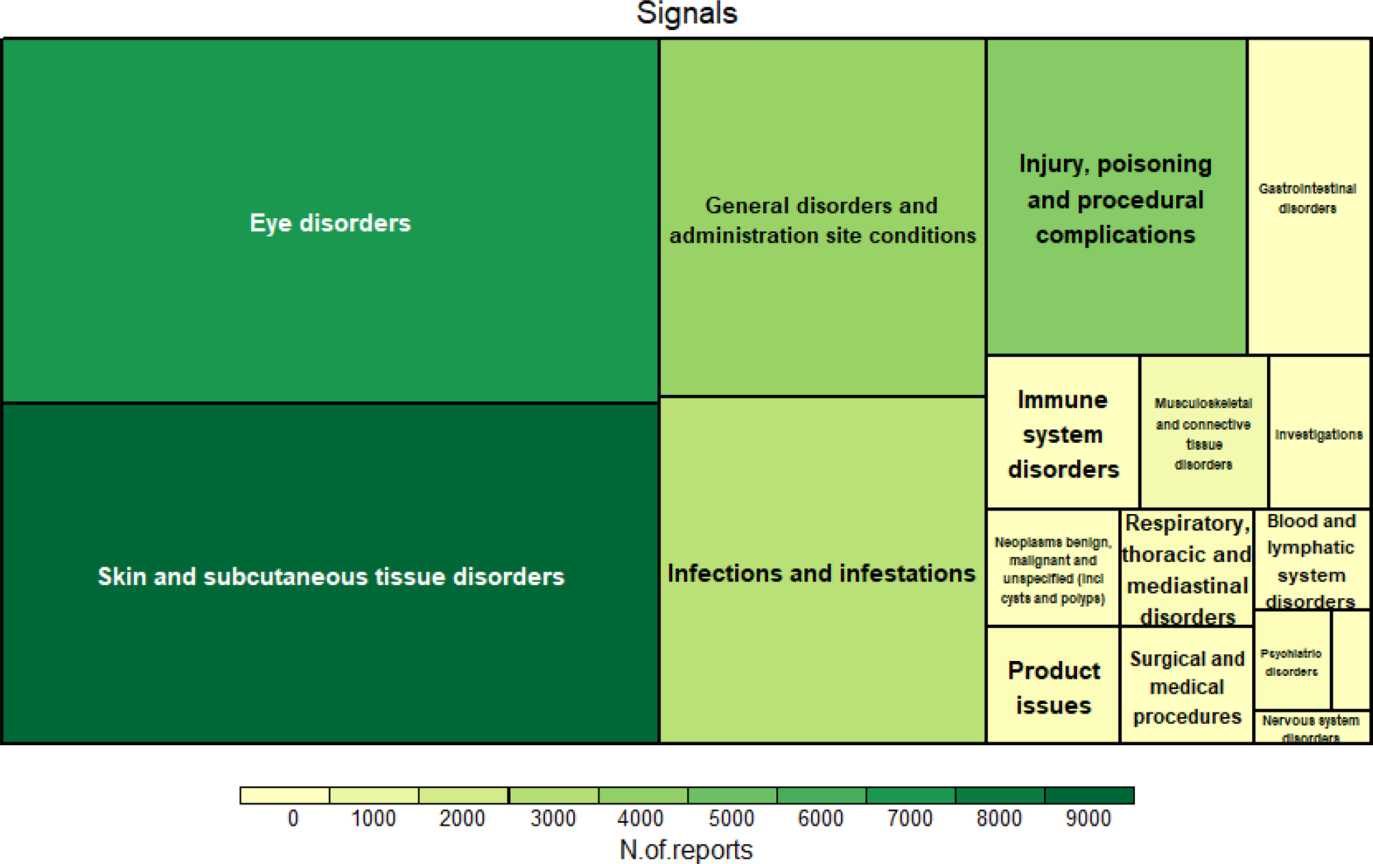
Tree map of dupilumab-related signals according to the system organ class (SOC) term.

A total of 246 signals in the PT level were detected for dupilumab. The signals classified in the primary SOC are shown in Fig. 1, and the tree maps of signals according to SOC, HLGT, and HLT are shown in Supplementary Fig. 1. Among the 246 positive signals, 61 were eye disorders, accounting for the largest percentage (24.8%), followed by skin and subcutaneous tissue disorders (23.17%); general disorders and administration site conditions (12.20%); infections and infestations (11.79%); injury, poisoning, and procedural complications (8.54%); and gastrointestinal disorders (4.07%). The number and percentage of signals and reports are shown in Supplementary Table 1.

### OSDs and dupilumab

Anatomically categorized eye disorder signals and their data mining indices are shown in Table 4.

**Table 4:** Anatomically classified dupilumab-related eye disorder signals.

Anatomical classification	Preferred term	Number of reports	PRR	ROR	IC_025_
Conjunctival	Conjunctivitis	1484	47.80	51.44	5.42
	Conjunctivitis allergic	51	45.71	45.82	4.53
	Conjunctivitis bacterial	17	104.73	104.82	3.92
	Noninfective conjunctivitis	4	137.18	137.21	1.34
	Conjunctival hyperemia	31	7.45	7.46	2.20
	Conjunctival irritation	4	15.36	15.36	0.82
	Conjunctival edema	6	3.85	3.85	0.28
	Seasonal allergy	36	6.39	6.40	2.06
Cornea	Keratitis	88	22.80	22.89	4.00
	Allergic keratitis	3	205.77	205.80	0.71
	Punctate keratitis	8	20.53	20.53	2.08
	Ulcerative keratitis	15	6.01	6.02	1.54
	Atopic keratoconjunctivitis	3	1543.27	1543.49	0.74
	Keratoconus	10	101.87	101.91	3.08
	Corneal degeneration	3	32.49	32.49	0.51
	Corneal disorder	5	3.94	3.94	0.11
	Corneal erosion	5	18.05	18.05	1.29
	Corneal scar	3	13.72	13.72	0.23
Lid, lash, and lacrimal	Eczema eyelids	18	71.78	71.84	3.85
	Erythema of eyelid	85	25.22	25.32	4.11
	Eyelid disorder	18	8.4	8.41	2.06
	Eyelid exfoliation	10	28.58	28.59	2.58
	Eyelid infection	3	11.83	11.83	0.16
	Eyelid irritation	48	43.13	43.23	4.43
	Eyelid margin crusting	78	78.37	78.66	5.31
	Eyelid pain	25	35.43	35.47	3.75
	Eyelid rash	18	51.73	51.77	3.68
	Eyelid skin dryness	41	166.73	167.06	5.25
	Eyelid thickening	5	29.74	29.74	1.5
	Eyelids pruritus	74	28.42	28.52	4.21
	Swelling of eyelid	75	20.05	20.12	3.79
	Ectropion	14	86.25	86.31	3.57
	Meibomian gland dysfunction	4	32.66	32.67	1.11
	Meibomianitis	3	40.08	40.09	0.55
	Blepharitis	168	55.99	56.44	5.3
	Chalazion	4	15.36	15.36	0.82
	Hordeolum	56	36.44	36.54	4.35
	Acquired dacryostenosis	11	15.29	15.3	2.25

## Discussion

In the present study, we found 61 signals associated with eye disorders, accounting for 25% of the positive signals, and 38 signals anatomically related to the ocular surface, including “conjunctival,” “corneal,” “lid,” “lash,” and “lacrimal”^28^. In previous phase 2b and phase 3 clinical trials that included patients with moderate to severe AD (SOLO1 and SOLO2), the dupilumab treatment groups had a greater incidence of conjunctivitis (7.3% in dupilumab 300 mg every week group and 9.7% in dupilumab 300 mg q2w group) than the placebo group (2.2%) after 16 weeks of treatment^29^. In a phase 3 clinical study (CHRONOS), the incidence rate of dupilumab-associated conjunctivitis was higher in patients with AD treated with topical corticosteroids than in patients in the placebo group (19.7% *vs*. 7.9%)^30^. However, in other clinical trials that included patients with moderate to severe uncontrolled asthma^31^, there was no difference between the dupilumab group and the placebo group with regard to the incidence of conjunctivitis and other eye disorders (2.3% *vs*. 3.3%). Upon integrated assessment of safety with phase 1–3 clinical studies, FDA-marked “conjunctivitis,” “blepharitis,” “keratitis,” “eye pruritus,” and “dry eye” were listed as the most common adverse reactions (incidence ≥ 1%) in the dupilumab drug label^32^. However, after updating the post-marketing safety information, a higher incidence (up to 28%) of dupilumab-related conjunctivitis was reported in several case series than in premarketing clinical trials, even though the number of cases was small^16,33^. Although there was a high incidence of conjunctivitis, post-marketing AE analysis showed that dupilumab-related eye disorders were not limited to “conjunctivitis,” which is consistent with the findings of the present study. Apart from “conjunctivitis,” a considerable number of case reports and case series have been published on dupilumab-induced OSDs^16^, including redness^33,34^, eyelid redness^35^, ectropion^35^, eyelid blisters^17^, eyelid swelling^36^, photophobia, dry eyes^17,33,37^, tearing^34^, blepharitis^17,37,38^, punctal stenosis^35,36^, periocular dermatitis^34^, and limbitis^38^, which is also consistent with our findings. Given that more diverse AEs associated with the ocular surface have been reported in the post-marketing setting, safety screening procedures should be broadened. It is difficult to identify the effects specific to dupilumab because many AEs are related to AD symptoms and to one another. However, it seems clear that dupilumab has various effects on the eye, although the underlying mechanisms are unclear.

Many hypotheses regarding the mechanism of dupilumab-related conjunctivitis have been proposed^39–42^. IL-13 inhibition has been suggested as the most plausible potential mechanism^40^. Tralokinumab and lebrikizumab, which are monoclonal antibodies against IL-13, were found to be associated with an increased risk of conjunctivitis in phase 3 and phase 2 clinical trials^43,44^. A significant association between IL-13 and increased human airway epithelia goblet cell (GC) density was also observed, as well as induced differentiation of GCs by IL-13^45–47^. GCs secrete gel-forming mucins and are distributed in the epithelium of the respiratory tract, gastrointestinal tract, and the conjunctiva^48^. In addition to eye disorders, mucous-related signals, including “oral mucosal erythema” and “oral mucosal blistering,” suggesting abnormal mucous function, were also detected in our study (Supplementary Table 2). Unlike in the respiratory and gastrointestinal tracts, the GCs in the conjunctiva are interspersed within a stratified epithelium^49^, implying that conjunctival GCs are more suggestible than other GCs. Conjunctival GCs play an important role in maintaining homeostasis of the ocular surface function^50^ by secreting mucins that lubricate and maintain surface wetting, thus, retaining the tear film across the epithelium, preventing infection, and removing debris from the ocular surface^51–53^. Several studies have shown that loss of conjunctival GCs occur in aqueous tear-deficient dry eye and ocular surface inflammatory diseases^54–56^. These findings suggest that the dysfunction of GCs caused by IL-13 blocking may be associated with an increased risk of OSDs.

A higher incidence of conjunctivitis (up to 18% in one clinical trial) was observed with dupilumab therapy than with tralokinumab (2–6%)^43^ and lebrikizumab (6–13%)^44^, suggesting that IL-4 plays a role in conjunctivitis. Although little is known about the effects of IL-4 on human conjunctival goblet cells, IL-4 has a direct effect on the differentiation of airway goblet cells from airway epithelial cells, increasing the expression of mucin gene and the production of mucous glycoconjugate^57^. Furthermore, IL-4Rα is abundantly expressed on the surface of the conjunctival epithelium^58^, indicating the potential effects of IL-4 blocking on the conjunctiva.

Although it appears that blocking IL-4 and IL-13 can trigger eye disorders, including conjunctivitis, there are still factors to consider about dupilumab-related eye disorders. In clinical trials, the incidence of dupilumab-related conjunctivitis was lower in patients with asthma or nasal polyposis (around 10%)^42,59^ than in patients with AD (up to 19.7%)^11,30^. Some features in patients with AD, such as eye involvement, may have contributed to eye disorders as a comorbidity of AD^42^. In addition, several risk factors, such as AD severity, high levels of thymus- and activation-regulated chemokines, IgE serum levels, circulating eosinophil counts, or a history of conjunctivitis may be responsible for conjunctivitis in patients with AD^40,59,60^. In particular, low serum levels of dupilumab seem to have an ordered relationship with a high incidence of conjunctivitis in groups divided by quartiles^59^. Additionally, patients with eye disorders showed lower drug efficacy, evaluated as the percentage change in eczema area and severity index and numeric rating scale outcomes, than those without eye disorders^61^. Therefore, the possibility of anti-drug antibody-related mechanisms could not be ruled out in dupilumab-induced eye disorders.

Spontaneous AE reports have some limitations, including underreporting and uncertainty of causality. Nevertheless, many unexpected AEs have been identified based on spontaneous AE reports. Although dupilumab-related eye disorders in patients with AD have been reported in previously published clinical trials for drug approval, the present study has some important highlights. First, our study possibly provides a better picture of data from premarketing clinical trials, additional case reports, and series. As real-world big data was used in the present study, it involved more comprehensive information and higher number of patient groups. Second, only conjunctivitis and keratitis were reported in the pre-market clinical trials, but our findings identified more diverse AEs associated with eye disorders through hierarchy analysis and anatomical classification (Table 4 and Supplementary Table 2). The 38 different ocular AEs are more diverse than those reported by any other research published thus far. In addition, the present study included reports from both case reports and case series and involved a wide range of content. From this point of view, our study provides important insights on safety issues related to the use of dupilumab. Further controlled and prospective studies are necessary to clarify the causality between dupilumab and various OSDs; however, our study provides a starting point for broad dupilumab safety screening in relation to eye disorders using real-world big data.

## Conclusions

Our results suggest that dupilumab causes extensive eye disorders, especially OSDs; however, the underlying mechanisms and risk factors remain unclear. Although further controlled and prospective studies are necessary to confirm the association between dupilumab and OSDs, the findings of this study may facilitate broader safety screening of dupilumab-related eye disorders based on real-world big data.

## Supplementary Information

Supplementary Information 1.

## Data Availability

The datasets analyzed are not publicly available because of ongoing data collection of adverse event reports but are available from UMC upon reasonable request.
