# Supplementary material for: Ocular surface disorders associated with the use of dupilumab based on WHO VigiBase
Source: Sci Rep. 2021 Jul 12;11:14293. doi: 10.1038/s41598-021-93750-3 (PMC8275737; doi:10.1038/s41598-021-93750-3)
Supplement: Supplementary file 1 — Supplementary Information 1. [file 41598_2021_93750_MOESM1_ESM.pdf]

Title: Ocular surface disorders associated with the use of dupilumab based on WHO VigiBase

Authors: Sunny Park, PhD[1], Jung Hyun Lee[2], Ji Hyun Park, PhD[3], So Hyang Park, BS[2], Song Yi Park, BS[2], Yong Woo Jung, PhD[1, 2], Soo An Choi, PhD[1, 2*]

[1]College of Pharmacy and Research Institute of Pharmaceutical Sciences, Korea University, Sejong, South Korea

[2]College of Pharmacy, Korea University, Sejong, South Korea
[3]College of Pharmacy, DukSung Women's University, Seoul, South Korea

Sunny Park and Jung-Hyun Lee contributed equally to this work.

***Corresponding author: Soo An Choi, PhD., Professor (sachoi@korea.ac.kr, +82-44-860-1626)**

ORCID: Sunny Park (0000-0001-9053-6930)

Jung Hyun Lee (0000-0001-5408-4965)

Ji Hyun Park (0000-0002-6705-2193)

So Hyang Park (0000-0001-6391-8298)

Song Yi Park (0000-0002-4697-5191)

Yong Woo Jung (0000-0002-5599-0553)

Soo An Choi (0000-0002-7831-7740)

Supplementary Table 1. Detected signals of dupilumab classified in primary SOC

System Organ Class (SOC)	No. of signals(%)	No. of reports(%)†
Eye disorders	61 (24.8)	6976 (24.3)
Skin and subcutaneous tissue disorders	57 (23.2)	8587 (29.9)
General disorders and administration site conditions	30 (12.2)	3928 (13.7)
Infections and infestations	29 (11.8)	2826 (9.8)
Injury, poisoning and procedural complications	21 (8.5)	4395 (15.3)
Gastrointestinal disorders	10 (4.1)	106 (0.4)
Immune system disorders	6 (2.4)	105 (0.4)
Musculoskeletal and connective tissue disorders	5 (2.0)	661 (2.3)
Investigations	4 (1.6)	65 (0.2)
Product issues	4 (1.6)	261 (0.9)
Neoplasms benign, malignant and unspecified (incl cysts and polyps)	4 (1.6)	43 (0.1)
Respiratory, thoracic and mediastinal disorders	4 (1.6)	326 (1.1)
Surgical and medical procedures	4 (1.6)	46 (0.2)
Blood and lymphatic system disorders	3 (1.2)	170 (0.6)
Psychiatric disorders	2 (0.8)	237 (0.8)
Metabolism and nutrition disorders	1 (0.4)	14 (0.0)
Nervous system disorders	1 (0.4)	4 (0.0)
Total	246	28750

† No. of reports means the number of reports of detected signals not including the negative adverse events.

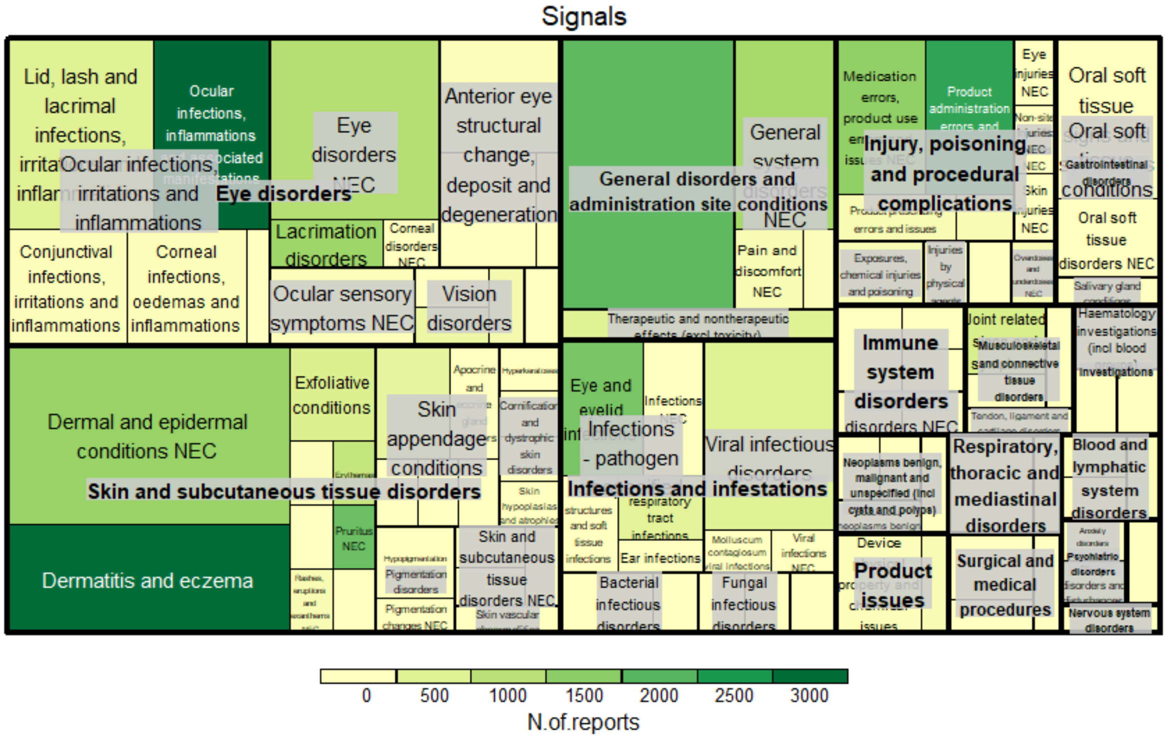

Supplementary Figure 1. Treemap of dupilumab related signals based on SOC, HLGT, and HLT terms

Supplementary Table 2. All detected signals and data mining indices in terms of primary SOC

PT	SOC	N. of reports	PRR	ROR	IC-2SD
**Eosinophilia**	Blood and lymphatic system disorders	74	3.57	3.58	1.46
**Lymphadenopathy**	Blood and lymphatic system disorders	92	2.03	2.03	0.70
**Lymphocytic infiltration**	Blood and lymphatic system disorders	4	13.11	13.11	0.74
**Abnormal sensation in eye**	Eye disorders	10	2.66	2.66	0.27
**Allergic keratitis**	Eye disorders	3	205.77	205.80	0.71
**Asthenopia**	Eye disorders	21	5.92	5.93	1.72
**Astigmatism**	Eye disorders	4	5.99	5.99	0.21
**Atopic keratoconjunctivitis**	Eye disorders	3	1543.27	1543.49	0.74
**Blepharitis**	Eye disorders	168	55.99	56.44	5.30
**Chalazion**	Eye disorders	4	15.36	15.36	0.82
**Conjunctival hyperaemia**	Eye disorders	31	7.45	7.46	2.20
**Conjunctival irritation**	Eye disorders	4	15.36	15.36	0.82
**Conjunctival oedema**	Eye disorders	6	3.85	3.85	0.28
**Conjunctivitis allergic**	Eye disorders	51	45.71	45.82	4.53
**Corneal degeneration**	Eye disorders	3	32.49	32.49	0.51
**Corneal disorder**	Eye disorders	5	3.94	3.94	0.11
**Corneal erosion**	Eye disorders	5	18.05	18.05	1.29
**Corneal scar**	Eye disorders	3	13.72	13.72	0.23
**Dacryostenosis acquired**	Eye disorders	11	15.29	15.30	2.25
**Dry eye**	Eye disorders	828	36.95	38.45	5.03
**Ectropion**	Eye disorders	14	86.25	86.31	3.57
**Eczema eyelids**	Eye disorders	18	71.78	71.84	3.85
**Episcleritis**	Eye disorders	7	17.70	17.70	1.80
**Erythema of eyelid**	Eye disorders	85	25.22	25.32	4.11
**Eye allergy**	Eye disorders	23	28.00	28.03	3.48
**Eye colour change**	Eye disorders	7	14.15	14.15	1.65
**Eye discharge**	Eye disorders	241	37.72	38.16	4.90
**Eye disorder**	Eye disorders	238	10.03	10.14	3.10
**Eye inflammation**	Eye disorders	122	22.96	23.09	4.10
**Eye irritation**	Eye disorders	635	18.38	18.94	4.04
**Eye oedema**	Eye disorders	12	5.02	5.02	1.18
**Eye pain**	Eye disorders	285	6.51	6.58	2.51
**Eye pruritus**	Eye disorders	1073	63.93	67.40	5.79
**Eye swelling**	Eye disorders	354	13.07	13.28	3.51

**Eye symptom**	Eye disorders	3	25.30	25.30	0.44
**Eye ulcer**	Eye disorders	8	47.03	47.05	2.48
**Eyelid disorder**	Eye disorders	18	8.40	8.41	2.06
**Eyelid exfoliation**	Eye disorders	10	28.58	28.59	2.58
**Eyelid irritation**	Eye disorders	48	43.13	43.23	4.43
**Eyelid margin crusting**	Eye disorders	78	78.37	78.66	5.31
**Eyelid pain**	Eye disorders	25	35.43	35.47	3.75
**Eyelid rash**	Eye disorders	18	51.73	51.77	3.68
**Eyelid skin dryness**	Eye disorders	41	166.73	167.06	5.25
**Eyelid thickening**	Eye disorders	5	29.74	29.74	1.50
**Eyelids pruritus**	Eye disorders	74	28.42	28.52	4.21
**Foreign body sensation in eyes**	Eye disorders	27	7.70	7.71	2.18
**Keratitis**	Eye disorders	88	22.80	22.89	4.00
**Keratoconus**	Eye disorders	10	101.87	101.91	3.08
**Lacrimation increased**	Eye disorders	501	25.99	26.62	4.50
**Limbal swelling**	Eye disorders	5	321.51	321.59	1.87
**Meibomian gland dysfunction**	Eye disorders	4	32.66	32.67	1.11
**Meibomianitis**	Eye disorders	3	40.08	40.09	0.55
**Noninfective conjunctivitis**	Eye disorders	4	137.18	137.21	1.34
**Ocular discomfort**	Eye disorders	30	6.14	6.15	1.93
**Ocular hyperaemia**	Eye disorders	957	27.36	28.65	4.63
**Ocular rosacea**	Eye disorders	5	72.45	72.47	1.73
**Periorbital swelling**	Eye disorders	12	3.97	3.97	0.89
**Photophobia**	Eye disorders	63	3.86	3.87	1.53
**Punctate keratitis**	Eye disorders	8	20.53	20.53	2.08
**Swelling of eyelid**	Eye disorders	75	20.05	20.12	3.79
**Ulcerative keratitis**	Eye disorders	15	6.01	6.02	1.54
**Vision blurred**	Eye disorders	308	2.94	2.97	1.38
**Visual impairment**	Eye disorders	240	2.07	2.09	0.86
**Vitreous floaters**	Eye disorders	19	3.80	3.80	1.10
**Chapped lips**	Gastrointestinal disorders	22	6.74	6.75	1.91
**Lip blister**	Gastrointestinal disorders	11	5.60	5.60	1.24
**Lip dry**	Gastrointestinal disorders	25	4.02	4.02	1.30
**Lip erythema**	Gastrointestinal disorders	6	12.65	12.65	1.35
**Lip exfoliation**	Gastrointestinal disorders	5	5.83	5.83	0.49
**Lip pain**	Gastrointestinal disorders	10	4.06	4.06	0.79
**Lip pruritus**	Gastrointestinal disorders	7	12.02	12.03	1.52
**Oral mucosal blistering**	Gastrointestinal disorders	11	2.16	2.16	0.06

**Oral mucosal erythema**	Gastrointestinal disorders	5	4.04	4.04	0.13
**Pigmentation lip**	Gastrointestinal disorders	4	48.99	49.00	1.21
**Condition aggravated**	General disorders and administration site conditions	795	5.11	5.27	2.24
**Discharge**	General disorders and administration site conditions	20	71.20	71.27	3.98
**Discomfort**	General disorders and administration site conditions	149	4.11	4.14	1.78
**Disease recurrence**	General disorders and administration site conditions	29	3.24	3.24	1.07
**Facial pain**	General disorders and administration site conditions	13	2.02	2.02	0.07
**Inflammation**	General disorders and administration site conditions	79	3.13	3.14	1.29
**Injection site bruising**	General disorders and administration site conditions	138	2.59	2.60	1.11
**Injection site coldness**	General disorders and administration site conditions	4	11.89	11.90	0.68
**Injection site discolouration**	General disorders and administration site conditions	32	3.12	3.13	1.05
**Injection site discomfort**	General disorders and administration site conditions	26	4.07	4.07	1.33
**Injection site dryness**	General disorders and administration site conditions	17	39.39	39.42	3.44
**Injection site eczema**	General disorders and administration site conditions	5	22.86	22.87	1.40
**Injection site exfoliation**	General disorders and administration site conditions	14	39.25	39.27	3.21
**Injection site irritation**	General disorders and administration site conditions	38	3.82	3.83	1.38
**Injection site mass**	General disorders and administration site conditions	188	5.14	5.18	2.13
**Injection site pain**	General disorders and administration site conditions	707	2.22	2.26	1.04
**Injection site paraesthesia**	General disorders and administration site conditions	9	6.95	6.95	1.30

**Injection site plaque**	General disorders and administration site conditions	3	10.22	10.22	0.09
**Injection site pruritus**	General disorders and administration site conditions	192	2.93	2.95	1.33
**Injection site rash**	General disorders and administration site conditions	153	4.68	4.71	1.97
**Injection site swelling**	General disorders and administration site conditions	291	2.48	2.50	1.14
**Injection site urticaria**	General disorders and administration site conditions	78	4.02	4.03	1.64
**Injection site vesicles**	General disorders and administration site conditions	16	2.47	2.47	0.45
**Secretion discharge**	General disorders and administration site conditions	27	4.99	5.00	1.62
**Swelling face**	General disorders and administration site conditions	117	2.26	2.27	0.89
**Symptom recurrence**	General disorders and administration site conditions	5	9.78	9.78	0.91
**Therapeutic response decreased**	General disorders and administration site conditions	406	5.02	5.10	2.17
**Therapeutic response shortened**	General disorders and administration site conditions	17	3.54	3.54	0.95
**Therapeutic response unexpected**	General disorders and administration site conditions	102	2.59	2.60	1.07
**Unevaluable event**	General disorders and administration site conditions	258	2.70	2.73	1.25
**Eosinophilic granulomatosis with polyangiitis**	Immune system disorders	7	6.51	6.51	0.99
**Food allergy**	Immune system disorders	13	4.29	4.30	1.04
**Seasonal allergy**	Immune system disorders	36	6.39	6.40	2.06
**Sensitisation**	Immune system disorders	3	14.70	14.70	0.26
**Serum sickness**	Immune system disorders	32	4.00	4.01	1.39
**Serum sickness-like reaction**	Immune system disorders	14	19.70	19.72	2.71
**Acarodermatitis**	Infections and infestations	5	8.56	8.56	0.81
**Conjunctivitis**	Infections and infestations	1484	47.80	51.44	5.42
**Conjunctivitis bacterial**	Infections and infestations	17	104.73	104.82	3.92
**Ear infection**	Infections and infestations	34	2.41	2.41	0.71
**Eczema herpeticum**	Infections and infestations	11	131.60	131.67	3.30

**Eye infection**	Infections and infestations	114	20.13	20.24	3.91
**Eye infection bacterial**	Infections and infestations	4	17.08	17.08	0.87
**Eye infection viral**	Infections and infestations	3	20.17	20.18	0.38
**Eyelid infection**	Infections and infestations	3	11.83	11.83	0.16
**Folliculitis**	Infections and infestations	15	4.05	4.05	1.06
**Fungal skin infection**	Infections and infestations	8	3.44	3.44	0.42
**Furuncle**	Infections and infestations	19	3.51	3.51	1.00
**Genital herpes**	Infections and infestations	7	6.64	6.65	1.01
**Herpes dermatitis**	Infections and infestations	3	25.72	25.72	0.45
**Herpes ophthalmic**	Infections and infestations	17	23.26	23.28	3.04
**Herpes simplex**	Infections and infestations	21	3.46	3.46	1.02
**Herpes virus infection**	Infections and infestations	24	6.29	6.29	1.86
**Herpes zoster**	Infections and infestations	105	2.08	2.08	0.76
**Hordeolum**	Infections and infestations	56	36.44	36.54	4.35
**Infection parasitic**	Infections and infestations	6	14.84	14.84	1.46
**Localised infection**	Infections and infestations	28	2.15	2.15	0.49
**Molluscum contagiosum**	Infections and infestations	17	53.32	53.37	3.62
**Nasopharyngitis**	Infections and infestations	307	2.66	2.68	1.24
**Ophthalmic herpes simplex**	Infections and infestations	8	37.58	37.60	2.39
**Oral herpes**	Infections and infestations	349	31.69	32.22	4.73
**Purulent discharge**	Infections and infestations	8	2.79	2.79	0.17
**Sinusitis**	Infections and infestations	124	2.13	2.14	0.82
**Skin infection**	Infections and infestations	23	4.06	4.06	1.28
**Tinea infection**	Infections and infestations	6	4.43	4.43	0.43
**Arthropod bite**	Injury, poisoning and procedural complications	11	2.65	2.65	0.33
**Device use issue**	Injury, poisoning and procedural complications	54	15.35	15.39	3.33
**Exposure during pregnancy**	Injury, poisoning and procedural complications	125	2.10	2.11	0.80
**Extra dose administered**	Injury, poisoning and procedural complications	90	5.76	5.78	2.17
**Incorrect dose administered**	Injury, poisoning and procedural complications	239	2.57	2.59	1.17
**Intentional dose omission**	Injury, poisoning and procedural complications	13	4.66	4.66	1.14
**Intercepted product prescribing error**	Injury, poisoning and procedural complications	6	7.67	7.67	0.96
**Lack of injection site rotation**	Injury, poisoning and procedural complications	4	6.24	6.25	0.24
**Paternal exposure timing unspecified**	Injury, poisoning and procedural complications	4	10.31	10.32	0.60
**Product administered to patient of inappropriate age**	Injury, poisoning and procedural complications	44	3.39	3.40	1.26
**Product dose omission**	Injury, poisoning and procedural complications	1914	9.49	10.37	3.17

**Product prescribing error**	Injury, poisoning and procedural complications	121	5.25	5.28	2.09
**Product storage error**	Injury, poisoning and procedural complications	125	2.45	2.45	1.02
**Product use in unapproved indication**	Injury, poisoning and procedural complications	348	5.35	5.42	2.25
**Product use issue**	Injury, poisoning and procedural complications	1053	12.98	13.63	3.58
**Scratch**	Injury, poisoning and procedural complications	52	9.06	9.08	2.64
**Sunburn**	Injury, poisoning and procedural complications	33	6.19	6.20	1.98
**Superficial injury of eye**	Injury, poisoning and procedural complications	11	21.81	21.82	2.53
**Underdose**	Injury, poisoning and procedural complications	138	3.29	3.30	1.45
**Unintentional use for unapproved indication**	Injury, poisoning and procedural complications	3	20.44	20.44	0.38
**Wrong schedule**	Injury, poisoning and procedural complications	7	20.40	20.41	1.88
**Blood immunoglobulin E increased**	Investigations	5	5.07	5.07	0.36
**Body temperature abnormal**	Investigations	4	5.05	5.05	0.06
**Eosinophil count abnormal**	Investigations	5	38.68	38.69	1.59
**Eosinophil count increased**	Investigations	51	12.95	12.98	3.10
**Alcohol intolerance**	Metabolism and nutrition disorders	14	9.18	9.19	1.98
**Arthralgia**	Musculoskeletal and connective tissue disorders	611	2.02	2.05	0.90
**Enthesopathy**	Musculoskeletal and connective tissue disorders	6	15.79	15.79	1.50
**Hip deformity**	Musculoskeletal and connective tissue disorders	3	16.16	16.16	0.30
**Joint stiffness**	Musculoskeletal and connective tissue disorders	38	2.12	2.12	0.57
**Seronegative arthritis**	Musculoskeletal and connective tissue disorders	3	16.68	16.69	0.31
**Cutaneous lymphoma**	Neoplasms benign, malignant and unspecified (incl cysts and polyps)	6	97.99	98.01	2.14
**Cutaneous T-cell lymphoma**	Neoplasms benign, malignant and unspecified (incl cysts and polyps)	12	16.27	16.28	2.39
**Skin papilloma**	Neoplasms benign, malignant and unspecified (incl cysts and polyps)	18	6.81	6.82	1.81
**T-cell lymphoma**	Neoplasms benign, malignant and unspecified (incl cysts and polyps)	7	12.35	12.36	1.55
**Sleep deficit**	Nervous system disorders	4	15.24	15.24	0.82
**Device defective**	Product issues	10	6.32	6.32	1.30
**Device issue**	Product issues	161	4.98	5.01	2.06
**Needle issue**	Product issues	78	9.88	9.92	2.88

**Syringe issue**	Product issues	12	2.43	2.43	0.27
**Fear of injection**	Psychiatric disorders	10	3.21	3.21	0.50
**Sleep disorder**	Psychiatric disorders	227	4.08	4.12	1.82
**Asthma**	Respiratory, thoracic and mediastinal disorders	167	3.17	3.18	1.42
**Oropharyngeal discomfort**	Respiratory, thoracic and mediastinal disorders	9	2.85	2.86	0.28
**Oropharyngeal pain**	Respiratory, thoracic and mediastinal disorders	139	2.03	2.03	0.76
**Upper-airway cough syndrome**	Respiratory, thoracic and mediastinal disorders	11	2.46	2.46	0.23
**Alopecia**	Skin and subcutaneous tissue disorders	377	2.26	2.28	1.02
**Alopecia areata**	Skin and subcutaneous tissue disorders	38	23.34	23.38	3.66
**Alopecia universalis**	Skin and subcutaneous tissue disorders	3	13.97	13.97	0.24
**Blister**	Skin and subcutaneous tissue disorders	105	2.23	2.23	0.86
**Dandruff**	Skin and subcutaneous tissue disorders	11	9.60	9.60	1.82
**Dermatitis**	Skin and subcutaneous tissue disorders	1091	27.63	29.12	4.65
**Dermatitis atopic**	Skin and subcutaneous tissue disorders	982	452.05	474.68	7.99
**Dermatitis exfoliative generalised**	Skin and subcutaneous tissue disorders	13	2.25	2.25	0.21
**Dry skin**	Skin and subcutaneous tissue disorders	674	10.14	10.45	3.21
**Eczema**	Skin and subcutaneous tissue disorders	746	25.21	26.12	4.49
**Eczema nummular**	Skin and subcutaneous tissue disorders	4	15.02	15.02	0.81
**Eczema weeping**	Skin and subcutaneous tissue disorders	3	22.21	22.21	0.41
**Erythema**	Skin and subcutaneous tissue disorders	898	3.14	3.23	1.55
**Erythema nodosum**	Skin and subcutaneous tissue disorders	13	2.45	2.45	0.32
**Exfoliative rash**	Skin and subcutaneous tissue disorders	13	4.50	4.50	1.10
**Hair growth abnormal**	Skin and subcutaneous tissue disorders	19	3.65	3.65	1.05
**Hyperkeratosis**	Skin and subcutaneous tissue disorders	12	3.15	3.16	0.61
**Lichenification**	Skin and subcutaneous tissue disorders	3	13.66	13.66	0.23
**Milia**	Skin and subcutaneous tissue disorders	6	20.37	20.38	1.65
**Miliaria**	Skin and subcutaneous tissue disorders	12	6.64	6.65	1.50
**Nail growth abnormal**	Skin and subcutaneous tissue disorders	6	8.18	8.18	1.02
**Neurodermatitis**	Skin and subcutaneous tissue disorders	18	19.68	19.70	2.95

**Pain of skin**	Skin and subcutaneous tissue disorders	47	3.12	3.12	1.16
**Papule**	Skin and subcutaneous tissue disorders	13	2.76	2.76	0.48
**Parakeratosis**	Skin and subcutaneous tissue disorders	6	53.68	53.69	2.02
**Perioral dermatitis**	Skin and subcutaneous tissue disorders	7	24.92	24.93	1.99
**Pigmentation disorder**	Skin and subcutaneous tissue disorders	31	6.16	6.17	1.95
**Pruritus**	Skin and subcutaneous tissue disorders	1801	2.07	2.18	0.98
**Rash macular**	Skin and subcutaneous tissue disorders	123	3.33	3.34	1.45
**Rebound atopic dermatitis**	Skin and subcutaneous tissue disorders	5	5144.22	5145.48	1.92
**Rebound eczema**	Skin and subcutaneous tissue disorders	19	2792.58	2795.16	4.49
**Rosacea**	Skin and subcutaneous tissue disorders	18	4.99	5.00	1.42
**Scab**	Skin and subcutaneous tissue disorders	39	4.86	4.87	1.72
**Seborrhoeic dermatitis**	Skin and subcutaneous tissue disorders	14	9.83	9.84	2.05
**Sensitive skin**	Skin and subcutaneous tissue disorders	11	11.83	11.83	2.02
**Skin atrophy**	Skin and subcutaneous tissue disorders	17	4.35	4.35	1.22
**Skin depigmentation**	Skin and subcutaneous tissue disorders	10	6.14	6.14	1.27
**Skin discolouration**	Skin and subcutaneous tissue disorders	153	3.58	3.60	1.59
**Skin discomfort**	Skin and subcutaneous tissue disorders	11	7.40	7.40	1.55
**Skin disorder**	Skin and subcutaneous tissue disorders	126	4.85	4.87	1.99
**Skin erosion**	Skin and subcutaneous tissue disorders	12	7.42	7.43	1.63
**Skin exfoliation**	Skin and subcutaneous tissue disorders	574	11.18	11.47	3.34
**Skin fissures**	Skin and subcutaneous tissue disorders	122	15.47	15.55	3.58
**Skin haemorrhage**	Skin and subcutaneous tissue disorders	54	9.24	9.27	2.68
**Skin hypertrophy**	Skin and subcutaneous tissue disorders	12	5.05	5.05	1.18
**Skin irritation**	Skin and subcutaneous tissue disorders	116	4.71	4.74	1.93
**Skin laxity**	Skin and subcutaneous tissue disorders	3	49.78	49.79	0.59
**Skin lesion**	Skin and subcutaneous tissue disorders	47	3.02	3.03	1.12
**Skin mass**	Skin and subcutaneous tissue disorders	39	5.07	5.08	1.78
**Skin plaque**	Skin and subcutaneous tissue disorders	19	4.45	4.46	1.31

**Skin sensitisation**	Skin and subcutaneous tissue disorders	5	7.21	7.22	0.67
**Skin swelling**	Skin and subcutaneous tissue disorders	30	6.67	6.68	2.04
**Skin texture abnormal**	Skin and subcutaneous tissue disorders	15	37.64	37.67	3.26
**Skin tightness**	Skin and subcutaneous tissue disorders	17	4.69	4.70	1.31
**Skin weeping**	Skin and subcutaneous tissue disorders	9	56.81	56.83	2.74
**Skin wrinkling**	Skin and subcutaneous tissue disorders	8	5.02	5.02	0.85
**Vitiligo**	Skin and subcutaneous tissue disorders	7	3.45	3.45	0.31
**Cataract operation**	Surgical and medical procedures	11	3.29	3.29	0.60
**Foot operation**	Surgical and medical procedures	7	3.35	3.35	0.27
**Hip arthroplasty**	Surgical and medical procedures	13	2.27	2.27	0.23
**Knee operation**	Surgical and medical procedures	15	4.15	4.15	1.09
